# Clinical prediction rules for the diagnosis of neuritis in leprosy

**DOI:** 10.1186/s12879-021-06545-2

**Published:** 2021-08-23

**Authors:** Louise Mara Giesel, Yara Hahr Marques Hökerberg, Izabela Jardim Rodrigues Pitta, Lígia Rocha Andrade, Debora Bartzen Moraes, José Augusto da Costa Nery, Euzenir Nunes Sarno, Marcia Rodrigues Jardim

**Affiliations:** 1 Leprosy Laboratory, Oswaldo Cruz Institute, Fiocruz, Av. Brasil, 4365, Manguinhos, Rio de Janeiro, RJ 21240-360 Brazil; 2 Post-Graduate Program in Neurology, Federal University of the State of Rio de Janeiro, Rio de Janeiro, RJ Brazil; 3 Laboratory of Clinical Epidemiology, Evandro Chagas National Institute of Infectious Diseases, Oswaldo Cruz, Brazil; 4 School of Medicine, Estácio de Sá University, Rio de Janeiro, Brazil; 5 Department of Neurology, Pedro Ernesto University Hospital/Rio de Janeiro State University, Rio de Janeiro, RJ Brazil

**Keywords:** Neuritis, Neuropathic pain, Leprosy, Clinical prediction rules, Sensitivity, Specificity

## Abstract

**Background:**

Diagnosing neuritis in leprosy patients with neuropathic pain or chronic neuropathy remains challenging since no specific laboratory or neurophysiological marker is available.

**Methods:**

In a cross-sectional study developed at a leprosy outpatient clinic in Rio de Janeiro, RJ, Brazil, 54 individuals complaining of neural pain (single or multiple sites) were classified into two groups (“neuropathic pain” or “neuritis”) by a neurological specialist in leprosy based on anamnesis together with clinical and electrophysiological examinations. A neurologist, blind to the pain diagnoses, interviewed and examined the participants using a standardized form that included clinical predictors, pain features, and neurological symptoms. The association between the clinical predictors and pain classifications was evaluated via the Pearson Chi-Square or Fisher’s exact test (p < 0.05).

**Results:**

Six clinical algorithms were generated to evaluate sensitivity and specificity, with 95% confidence intervals, for clinical predictors statistically associated with neuritis. The most conclusive clinical algorithm was: pain onset at any time during the previous 90 days, or in association with the initiation of neurological symptoms during the prior 30-day period, necessarily associated with the worsening of pain upon movement and nerve palpation, with 94% of specificity and 35% of sensitivity.

**Conclusion:**

This algorithm could help physicians confirm neuritis in leprosy patients with neural pain, particularly in primary health care units with no access to neurologists or electrophysiological tests.

## Background

In leprosy, diagnosing neural pain can be difficult, especially for a non-specialist. The differential diagnosis between neuritis and neuropathic pain is critical because these involve distinct pathological processes requiring different treatments [4]. Misdiagnosis tends to result in prescription errors, as has been frequently identified by specialists of reference centers receiving patients forwarded from primary health care units. Inadequate treatment then leads to unfavorable prognosis, particularly with regards to the prescription of corticosteroids, which may not be prescribed as necessary for neuritis or may be inappropriately used for cases of neuropathic pain [14, 21].

Neuritic pain starts with an inflammatory process affecting nerves. Neuropathic pain, a nonexclusive condition of leprosy, begins because of abnormal functioning of the peripheral and central nervous systems. Neuritis and neuropathic pain are commonly confused with each other. Thus, it is difficult to reach a correct differential diagnosis and prescribe adequate treatment when both conditions occur concomitantly [3, 8, 23].

In leprosy, the frequency of neuropathic pain ranges from 11.3 to 70.3% [12, 17, 20, 27]. In our Leprosy Outpatient Clinic based in Rio de Janeiro, the annual prevalence is 15% [9]. Although sensitive questionnaires are currently available to identify neuropathic pain, like the Douleur Neuropathique en 4 Questions (DN4), due to a low specificity rate (45–57.9%), it is not considered adequate [12, 16].

The lack of diagnostic tests has hampered the diagnosis of reactional episodes like reversal reaction or erythema nodosum leprosum. Besides this, there are no laboratory or neurophysiological tools able to identify neuritis [2]. Therefore, the purpose of this work was to define a simple tool to diagnose neuritis in leprosy patients and identify important clinical predictors to assist this.

The present study evaluated clinical predictors used to differentiate neuritis from neuropathic pain in leprosy neuropathy patients via a standardized form that included subjective questions and physical examination data. Few health units employ specialists in neurology or have resources for electrophysiological evaluations that could facilitate diagnosing patients with chronic neuropathic pain. Therefore, defining clinical predictors to diagnose neuritis could become a diagnostic tool to help clarify uncertain cases.

## Methods

### Subjects

The present cross-sectional observational study was carried out at the Souza Araújo Outpatient Clinic, a national referral center for the diagnosis and treatment of leprosy at the Oswaldo Cruz Foundation (Fiocruz), under the Brazilian Ministry of Health, in Rio de Janeiro, RJ, Brazil, from September 2014 to May 2017. Eligibility criteria included leprosy patients with neural pain in any limb, except for cranial nerves, at least 18 years of age, fluency in the Portuguese language, and ability to understand questions during anamnesis. Exclusion criteria were the presence of other neuropathic pain etiologies such as human immunodeficiency virus infection and chronic diseases; pain syndromes like complex regional syndromes, fibromyalgia, painful ulcerations, radiculopathy, joint pain, and tendonitis; and patients that had been undergoing corticosteroid therapy (more than 10 mg of prednisone or equivalence).

### Clinical history and examination

After obtaining the written informed consent of the participants, information regarding sex, age, educational level, and the World Health Organization (WHO) leprosy classification were recorded. Neurological evaluations focusing on the peripheral nerves were performed and patients with more than one affected limb were asked to answer separately to each of them. The main leprosy-affected peripheral nerves (superior auricular, ulnar, radial cutaneous, lateral popliteal, and posterior tibial nerves) were assessed for enlargement [6]; and palms and soles were examined for the presence of cyanosis. In brief, the tactile threshold was tested with Semmes–Weinstein monofilaments [25]. The monofilaments vary in thickness (1 = 300 g, 2 = 4 g, 3 = 2 g, 4 = 0.2 g, and 5 = 0.05 g); and the inability to perceive the touch of even one of them represents an absence of tactile sensitivity to that given pressure. Thermal sensation was determined by the use of cold (15 °C) metal objects; and a safety pin was utilized to ascertain pain perception in the trigeminal, ulnar, median, radial, sural, superficial fibular, and plantar bilateral nerves. Lastly, the Medical Research Council (MRC) scale was adopted to determine individual muscle strength in the upper and lower limbs; and tendon reflexes were tested using Taylor’s hammer [13].

### Standardized form

A standardized form was developed (Table 1) consisting of the following items: date of onset of pain, sensory and motor symptoms, worsening factors, irradiation signs, and pain triggered by nerve palpation, whether declared spontaneously or after questioning [6, 9, 27]. Items were subsequently combined to create the following three subgroups: (a) ‘sensory symptoms’ for positivity in questions 2 or 3; (b) ‘motor symptoms’ for positivity in questions 4 or 5; and (c) ‘neurological symptoms’ for those who answered positively to any of the above questions, 2, 3, 4, or 5 (Table 1). Finally, a pre-test was conducted with ten patients to evaluate the comprehension of the standardized form and their response time.

**Table 1:** Preliminary questionnaire items

Subjective questions			
1. When did your pain start?	Less than 30 days	30–90 days	More than 90 days
2. Do you feel numbness or diminished skin tone in the same area that feels pain?	No	Yes, until 30 days	Yes, 30 days or more
3. Do you feel tingling in the same area that feels pain?	No	Yes, until 30 days	Yes, 30 days or more
4. Do you feel weakness in the hand and arm or foot and leg that feels pain?	No	Yes, until 30 days	Yes, 30 days or more
5. Have you noticed frequent trouble opening screw bottles or lock with key?/Have you noticed frequent tripping or difficulty wearing flip-flops or sandals?	No	Yes, until 30 days	Yes, 30 days or more
6. You notice pain worsening	Waking up in the morning	No	Yes
	When you go bed at night	No	Yes
	Making movements with hands or arms/foots and legs	No	Yes
	After effort, carry weight or perform manual work	No	Yes
Physical examination			
7. Examine radial, median and ulnar nerves/fibular, sural and tibial nerves through palpation. Ask patient about trigger pain by nerve palpation	Pain is palpation spontaneously declared bfore question	Pain is referred after question	Patient negative trigger pain by nerve palpation
8. Ask patient to locate the pain area. Is there pain irradiation when describing area affected?	No	Yes	
9. How you think about these questions	Easy to answer	Difficult to answer	Indifferent and had no opinion about it

One neurologist, unaware of the previous neural pain diagnoses, independently evaluated recruited participants during a routine visit via the standardized form. Patients with more than one affected limb were instructed to complete a form for each limb separately.

### Electrophysiological examination

Nerve conduction was verified in the painful limbs of all participants. Parameters were measured by way of the Neuropack μ MEB 9100 EP/EMG measuring system (Nihon Kohden Corp., Tokyo, Japan). Skin temperatures were taken at the wrists and ankles and maintained above 33 °C in room temperatures ranging from 29 to 32 °C. Standard methods were performed according to Delisa, 1994 [5]. Sensory nerve conduction studies included the radial, median, ulnar, sural, and superficial fibular sensory nerves. Motor nerve conduction studies were performed on the ulnar, median, tibial, and common peroneal nerves [28].

### Case definitions

Based on the patient history and examination results, patients were classified into two diagnostic groups by a neurologist specialized in leprosy: *neuropathic pain* and *neuritis* (Table 2). The diagnostic criteria for neuropathic pain was: pain distribution in a neuro-anatomically plausible area with confirmed negative or positive sensory signs (i.e., hypoesthesia, hyperesthesia, hypoalgesia, hyperalgesia, or allodynia) [11]. The definition for neuritis was: pain in the neuro-anatomical area but in conjunction with motor impairment and/or sensory signs in the correspondent nerve, in addition to confirmed demyelinating signs demonstrated in the electrophysiological examination results [15]. Demyelinating signs are defined as such when there is a latency prolongation of the compound muscle action potential (CMAP) and/or sensory nerve action potential (SNAP); reduction of the motor or muscular conduction velocity to below 85% of the lower limit of normality circumventing the CMAP; or a SNAP amplitude drop by up to 30% of the lower limit [1, 7, 13]. If the patients with demyelinating signs had been treated for neuritis in the previous year, they were assigned to the neuropathic pain group. Despite the paucity of data in the medical literature regarding a gold standard to define neuritis, a satisfactory response of up to one month of corticoid therapy was used for diagnostic confirmation.

**Table 2:** Diagnostic criteria for neuropathic pain and neuritis

Case definitions	
Neuropathic pain	Neuritis
1. Pain with a distinct neuroanatomically plausible distribution, with motor impairment and/or sensory signs in the correspondent nerve; 2. Demonstration of any neural lesion in the electrophysiological examination results in correspondent nerve of pain area, except with demyelinating electrophysiological signs with criteria for neuritis	1. Neural pain in conjunction with motor impairment and/or sensory signs in the correspondent nerve; 2. Demonstration of neural lesion with demyelinating signs in the electrophysiological examination results, in correspondent nerve of pain area; If patients had already treated for neuritis in correspondent nerve of pain area previously, they were allocated to the neuropathic pain group 3. Satisfactory response of up to one month of corticoid therapy (pain relief or clinical improve)

### Statistical analysis

The data were analyzed via SPSS, version 16 [26]. The sample demographic and clinical features were described using median and interquartile interval for both nonparametric quantitative variables and frequencies as well as proportions of the categorical variables. Pearson Chi-Square and Fisher’s exact test were adopted to assess the association between demographic and clinical predictors. The diagnosis of neuritis or neuropathic pain was determined by the results of the neurological and electrophysiological examinations at a significance level of p < 0.05.

The minimum estimated sample size was 57 neural pain evaluations to obtain 80% of sensitivity and 34 for 90% of specificity, considering an acceptable difference of 10% and a 95% confidence level in an estimated population of 750 patients with neural pain.

Clinical algorithms were built that could predict neuritis using the clinical predictors statistically associated with neuritis in analysis and the ones based on the rationale of clinical practice. For each algorithm, indicators of test accuracy were estimated with 95% confidence intervals.

## Results

Of the 161 eligible patients, 107 were excluded. Sixteen were incompatible because of diabetic neuropathies and 5 due to other neuropathies. Fifty-one were in already in treatment for neuropathic pain, 15 for neuritis, and 22 were in corticosteroid therapy. Sixteen had other pain conditions and 3 were excluded for other causes. The final sample included 54 patients with a total of 124 completed forms. Fourteen patients had neuritis, six of which had neural pain in more than one limb and were simultaneously diagnosed with neuritis and neuropathic pain; five had neuritis in only one affected limb and three in two affected limbs (Table 3); therefore, a total of 20 forms (16.1%) were described as neuritis. Demographic characteristics of the diagnostic groups are presented in Tables 4, 5. The median age of the study sample was 44. The majority had two or more painful neural sites, roughly four years of formal education, and a WHO multibacillary leprosy classification.

**Table 3:** Description of neuritis in 20 limbs of 14 patients

Patient	N° affected limbs (upper/lower)	Limb with Neuritis (n = 20)	Pain onset (days)	Motor impairment	Triggered by nerve palpation	Sensory signs
1	4 (2/2)	Upper	> 90	> 30 days	Spontaneously	> 30 days
		Upper	15 |‒30	No	Spontaneously	≤ 30 days
2	2 (1/1)	Upper	60 |‒90	> 30 days	Spontaneously	> 30 days
3	1 (1/0)	Upper	7 |‒15	≤ 30 days	When asking	> 30 days
4	2 (2/0)	Upper	> 90	> 30 days	When asking	> 30 days
		Upper	> 90	> 30 days	When asking	> 30 days
5	3 (1/2)	Upper	> 90	> 30 days	Spontaneously	> 30 days
6	1 (1/0)	Upper	7 |‒15	> 30 days	No pain	> 30 days
7	2 (0/2)	Lower	< 7	No	When asking	No
		Lower	< 7	No	When asking	No
8	2 (2/0)	Upper	> 90	No	Spontaneously	> 30 days
9	3 (1/2)	Upper	> 90	No	No pain	No
		Lower	> 90	> 30 days	No pain	> 30 days
10	1 (0/1)	Lower	> 90	> 30 days	When asking	> 30 days
11	2 (2/0)	Upper	60 |–90	> 30 days	Spontaneously	> 30 days
		Upper	60 |–90	> 30 days	Spontaneously	> 30 days
12	4 (2/2)	Lower	> 90	> 30 days	Spontaneously	> 30 days
		Lower	> 90	> 30 days	Spontaneously	> 30 days
13	1 (1/0)	Upper	30 |–60	> 30 days	No pain	> 30 days
14	1 (1/0)	Upper	30 |–60	> 30 days	When asking	> 30 days

**Table 4:** Demographic and clinical characteristics of 54 leprosy patients (2014–2017) with neural pain

Variables	N (%)
Sex	
Male	33 (61.1)
Female	21 (38.9)
Age	
Median (IQI)	44.0 (32.0–61.75)
Skin colour	
White	16 (29.6)
Black	11 (20.4)
Other	8 (14.8)
Years of schooling	
< 8 years	24 (44.4)
8–11 years	13 (24.1)
> 11 years	17 (31.5)
WHO classification	
PB	12 (22.2)
MB	42 (77.8)
Number of available limbs with painful area for each patient	
1	13 (24.1)
2	23 (42.6)
3	7 (13.0)
4	11 (20.4)
Interview response time in minutes (SD)	3.7 (1.12)
Time of leprosy onset in years	
Median (IQI)	3.0 (1.0–5.0)

*IQI* interquartile interval, *PB* paucibacillary, *MB* multibacilary, *SD* standard deviation

**Table 5:** Frequency of gender and site of neural pain described on 124 forms submitted by 54 leprosy patients with neural pain

Variable	Neuritis N (%)	Neuropathic pain N (%)	Total N (%)	p-Value^1^
Gender				
Male	14 (70.0)	61 (58.7)	75 (60.5)	0.3418^1^
Female	6 (30.0)	43 (41.3)	49 (39.5)	
Limb				
Upper	14 (70.0)	38 (36.5)	52 (41.9)	0.0055^1^
Lower	6 (30.0)	66 (63.5)	72 (58.1)	

^1^Pearson Chi-square test

Significant differences were found between the diagnostic groups (“neuropathic pain” and “neuritis”) with regards to when the pain began; if the pain had lasted up to 90 days (p = 0.031); if movement was a worsening pain factor (p = 0.042); and if the pain was triggered by nerve palpation (p = 0.039). The set of analyzed questions and answers is presented in Table 6.

**Table 6:** Clinical predictors of *neuritis* and *neuropathic pain* (n = 124)

Variable	Neuritis N (%)	Neuropathic pain N (%)	p-Value^1^
Date of pain onset (2 categories)			
Up to 90 days ago	10 (50.0)	27 (26.0)	0.031
> 90 days	10 (50.0)	77 (74.0)	
Sense of numbness			
No	7 (35.0)	22 (21.2)	0.191^2^
Yes (< 30 days)	2 (10.0)	5 (4.8)	
Yes (≥ 30 days)	11 (55.0)	77 (74.0)	
Sense of paraesthesia			
No	5 (25.0)	29 (27.9)	0.454^2^
Yes (< 30 days)	2 (10.0)	4 (3.8)	
Yes (≥ 30 days)	13 (65.0)	71 (68.3)	
Sense of weakness			
No	9 (45.0)	33 (31.7)	0.263^2^
Yes (< 30 days)	2 (10.0)	6 (5.8)	
Yes (≥ 30 days)	9 (45.0)	65 (62.5)	
Motor difficulties			
No	5 (25.0)	26 (25.0)	0.706^2^
Yes (< 30 days)	1 (5.0)	3 (2.9)	
Yes (≥ 30 days)	14 (70.0)	75 (72.1)	
Sensory symptoms			
No	3 (15.0)	15 (14.4)	0.472^2^
Yes (< 30 days)	1 (5.0)	4 (3.8)	
Yes (≥ 30 days)	16 (80.0)	85 (81.7)	
Motor symptoms			
No	5 (25.0)	13 (12.5)	0.277^2^
Yes (< 30 days)	1 (5.0)	5 (4.8)	
Yes (≥ 30 days)	14 (70.0)	86 (82.7)	
Neurological symptoms			
No	3 (15.0)	3 (2.9)	0.103^2^
Yes (< 30 days)	1 (5.0)	5 (4.8)	
Yes (≥ 30 days)	16 (80.0)	96 (92.3)	
Worse in the morning			
No	11 (55.0)	64 (61.5)	0.584
Yes	9 (45.0)	40 (38.5)	
Worse at night			
No	7 (35.0)	44 (42.3)	0.543
Yes	13 (65.0)	60 (57.7)	
Worse upon movement			
No	6 (30.0)	57 (54.8)	0.042
Yes	14 (70.0)	47 (45.2)	
Worse with physical exertion			
No	6 (30.0)	35 (33.7)	0.750
Yes	14 (70.0)	69 (66.3)	
Triggered by nerve palpation			
No	4 (20.0)	53 (51.0)	0.039
Yes, spontaneously declared	9 (45.0)	28 (26.9)	
Yes, declared after questioning	7 (35.0)	23 (22.1)	
Irradiated pain			
No	6 (30.0)	37 (35.6)	0.631
Yes	14 (70.0)	67 (64.4)	

^1^Pearson Chi-square test; ^2^Fisher’s exact test

The presence of any sensory or motor symptoms for at least 30 days was not found to be statistically different between the two groups (p = 0.103). However, this is a common clinical finding during routine evaluations of neuritis, and it had been chosen to discriminate between neuritis and neuropathic pain.

**Fig. 1:**
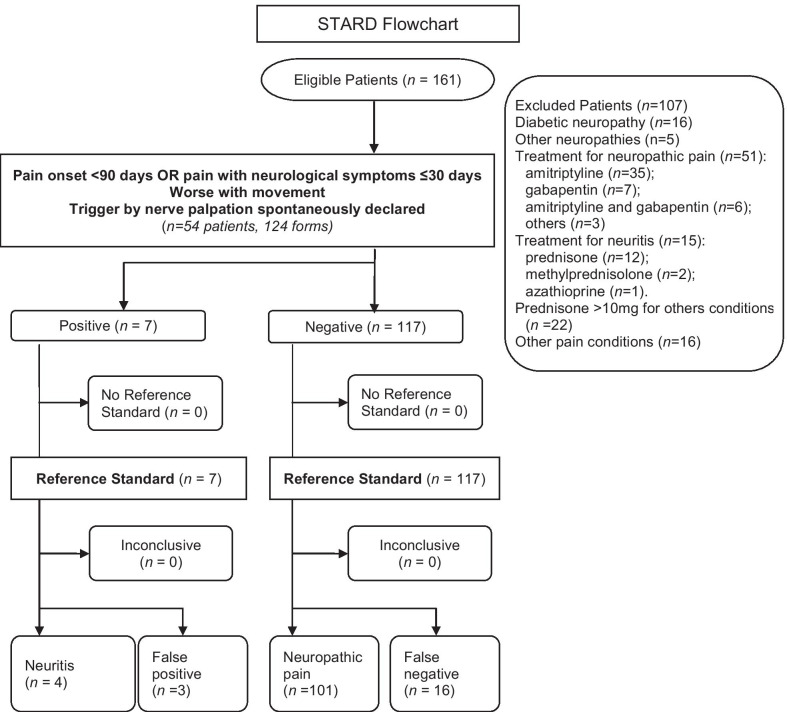
Standards for the Reporting of Diagnostic Accuracy (STARD) Flowchart for the diagnosis of neuritis

Algorithms based on the clinical predictors identified on the forms were used to diagnose the subtype of neural pain (Table 7). All algorithms tested are presented in Table 7. Pain onset in the last 90 days or pain with any of the typical symptoms for at least 30 days, in association with movement and spontaneously-declared triggering of pain by palpation as factors in the worsening of pain, were analyzed as diagnostic algorithms for neuritis, showing a negative predictive value of 86.3%, a specificity of 97.12%, and a positive likelihood ratio of 6.93. When considering pain triggered by palpation in any condition (spontaneously referred or not), sensitivity increased from 20.0 to 35.0%, so that, as described in Fig. 1, a diagnosis of “confirmed neuritis” could be reached.

**Table 7:** Accuracy of six clinical algorithms for the diagnosis of neuritis in leprosy patients with neural pain (n = 124)

Clinical algorithms	Se % (95% CI)	Sp % (95% CI)	PPV % (95% CI)	NPV % (95% CI)	PLR % (95% CI)	NLR % (95% CI)	DOR (95% CI)
Pain onset < 90 days OR pain with neurological symptoms ≤ 30 days AND movement as worsening pain factor AND spontaneously-declared triggering of pain by palpation	20.00 (8.07–41.60)	97.12 (91.86–99.01)	57.1 (26.02–83.48)	86.3 (83.35–88.84)	6.93 (1.68–28.64)	0.82 (0.66–1.03)	8.42 (1.72–41.16)
Pain onset < 90 days OR pain with neurological symptoms ≤ 30 days AND movement as worsening pain factor AND triggering of pain by palpation	35.00 (18.12–56.71)	94.23 (87.98–97.33)	53.8 (31.06–75.13)	88.30 (84.42–91.30)	6.07 (2.28–16.16)	0.69 (0.50–0.95)	8.80 (2.56–30.22)
Pain onset < 90 days OR pain with neurological symptoms ≤ 30 days AND spontaneously-declared triggering of pain by palpation	20.00 (8.07–41.60)	91.35 (8.37–95.38)	30.80 (13.61–55.63)	85.60 (82.41–88.27)	2.31 (0.79–6.78)	0.88 (0.70–1.10)	2.64 (0.72–9.60)
Pain onset < 90 days OR pain with neurological symptoms ≤ 30 days AND triggering of pain by palpation	40.0 (21.88–61.34)	86.54 (78.66–91.81)	36.4 (21.83–53.91)	88.20 (83.83–91.56)	2.97 (1.44–6.13)	0.69 (0.48–1.0)	4.29 (1.49–12.33)
Pain onset < 90 days OR pain with neurological symptoms ≤ 30 days AND physical exertion and movement as worsening pain factors AND spontaneously-declared triggering of pain by palpation	20.00 (8.07–41.60)	95.19 (89.24–97.93)	44.4 (19.87–72.08)	86.1 (83.05–88.65)	4.16 (1.22–14.16)	0.84 (0.67–1.05)	4,95 (1.20–20.42)
Pain onset < 90 days OR pain with neurological symptoms ≤ 30 days AND physical exertion and movement as worsening pain factors AND triggering of pain by palpation	35.0 (18.12–56.71)	91.35 (84.37–95.38)	43.7 (25.01–64.46)	88.0 (83.98–91.06)	4.04 (1.70–9.60)	0.71 (0.51–0.99)	5.68 (1.81–17.86)

*Se* sensitivity, *Sp* specificity, *PPV* positive predictive value, *NPV* negative predictive value, *PLR* positive likelihood ratio, *NLR* negative likelihood ratio, *DOR* diagnostic odds ratio, *95% CI* 95% confidence interval

Most of the patients assessed considered the form easy to answer (68.5%), while one (1.9%) found it difficult, 22.2% were indifferent, and 3.7% had no opinion.

## Discussion

Neural pain is a common complaint during medical appointments with leprosy patients**.** Neuropathy in leprosy starts with the entry of *Mycobacterium leprae* in the Schwann cells of the small nerve fibers [18, 19]. However, acute and subacute neural damage may also occur due to neuritis and reactional episodes, characterized by the mixture of neurological symptoms with neural pain. In the acute phases, the diagnosis of neuritis is relatively clear. In the later stage, characterized by late nerve impairments due to intra-neural fibrosis, reactional episodes may reoccur, further complicating an accurate diagnosis of neuritis, especially in patients with neuropathic pain or chronic neuropathy [7, 22, 23].

Anamnesis is essential in the differential diagnosis of neural pain. Locating the onset of pain and neurological symptoms might be one of the most effective diagnostic indicators at our disposal. As expected, the persistence of pain beyond 90 days was more frequent in cases of neuropathic pain, with a statistically significant difference when comparing these cases to those with neuritis. In contrast, identifying movement as a factor in worsening pain might not seem specific enough, yet, there is a statistically significant difference between the diagnostic groups regarding this factor. Moreover, in the absence of this information, positive likelihood ratios decreased in the neuritic diagnostic algorithm.

Neurological evaluations focusing on the peripheral nerves and their enlargement plus trigger pain by nerve palpation were the clinical signs that best predicted neuritis. However, isolated thickening of the peripheral nerves should not be the only support for its diagnosis. Knowledge of overall anatomy and the use of peripheral nerve palpation in leprosy are crucial in researching identifying potential trigger points for pain and should be encouraged in clinical practice [7].

Pain triggered by nerve palpation raises the possibility of an inflammatory component in the pathophysiological process of pain, which, due to local inflammation, would naturally be present in neuritis [9]. This finding was common in these cases. This isolated sign did not reach an acceptable level of accuracy, primarily because it is present in almost half of all patients with neuropathic pain and because dysesthesia upon nerve palpation can occur in many reactional states [9, 15].

There were limitations to calculate sample size considering divergence register about acute neuritis incidence. Datas were based on cohorts that estimate the incidence of neuritis in leprosy reactions, without differentiating neuritis with or without pain [10, 24]. The term “neuritis” in the medical literature has been used for different contexts, both in the pathophysiological process of nerve involvement in leprosy and in the clinical diagnosis of the leprosy reaction. There is not a clear and reliable gold standard for neuritis diagnosis.

During case definition, defining the diagnostic criteria for neuritis was more difficult than for neuropathic pain [11]. It is well known that leprosy is first a demyelinating neuropathy, which, as it progresses, leads to axonal loss [7]. The present study incorporated the neurophysiological criteria for demyelination that have been used in clinical practice by highly reliable reference centers to reach a definitive diagnosis of neuritis, despite the absence of these data in the medical literature. Therefore, pain relief following corticoid therapy was included as a confirmatory diagnostic. We believe that the criteria to define neuritis, as presented here, could be a method used by other researchers to diagnose this condition.

Date of pain onset at any time during the 90-day period prior to examination or the simultaneous occurrence of pain and neurological symptoms within the last 30 days, in association with the spontaneously-declared triggering of pain by nerve palpation and movement as factors of pain worsening showed higher positive likelihood ratios, resulting in an increased specificity in the diagnosis of neuritis. However, when nerve palpation alone triggered pain, whether spontaneously declared or after questioning the patient, both the specificity and positive likelihood ratios decreased, albeit only slightly for the latter.

## Conclusion

In conclusion, the present study determined that the sum of the following data satisfactorily confirmed a diagnosis of neuritis: the occurrence of the onset of pain at any time within the previous 90 days or in association with the initiation of neurological symptoms sometime during the prior 30-day period, plus the worsening of pain as a result of movement and nerve palpation. When a negative result is obtained, individuals without neuritis are correctly identified, enabling the appropriate medications to be prescribed. This avoids the indiscriminate use of corticosteroids, a common problem seen by reference centers, especially in patients with neuropathic pain or chronic neuropathy.

Perhaps even more importantly, the results of the present study unequivocally show that a correct diagnosis could be reached by (1) a non-specialist and (2) without any neurophysiological testing whatsoever.

There is hope that these findings assure more frequent and reliable diagnoses of this particularly neglected disease and greatly facilitate its case management in primary health care units with limited resources.

## Data Availability

The datasets used and/or analyzed during the current study are available from the corresponding author on reasonable request.

## Abbreviations

CMAP: Compound muscle action potential
DN4: Douleur Neuropathique en 4 Questions
Fiocruz: Oswaldo Cruz Foundation
MRC: Medical Research Council
SNAP: Sensory nerve action potential
WHO: World Health Organization

## References

[1] Andrade PR, Jardim MR, da Silva AC, Manhaes P, Antunes SL, Vital RT, Prata RB, Petito RB, Pinheiro RO, Sarno EN (2016). Inflammatory cytokines are involved in focal demyelination in leprosy neuritis. J Neuropathol Exp Neurol.

[2] Antunes DE, Ferreira GP, Nicchio MV, Araujo S, Cunha AC, Gomes RR, Costa AV, Goulart IM (2016). Number of leprosy reactions during treatment: clinical correlations and laboratory diagnosis. Rev Soc Bras Med Trop.

[3] Arco RD, Nardi SM, Bassi TG, Paschoal VD (2016). Diagnosis and medical treatment of neuropathic pain in leprosy. Rev Lat Am Enfermagem.

[4] Cohen SP, Jianren M (2014). Neuropathic pain: mechanisms and their clinical implications. BMJ.

[5] Delisa JA, Lee HJ, Baran EM (1994). Manual of nerve conduction velocity and clinical neurophysiology.

[6] Elvey RL (1997). Physical evaluation of the peripheral nervous system in disorders of pain and dysfunction. J Hand Ther.

[7] Garbino JA, Heise CO, Marques WJ (2016). Assessing nerves in leprosy. Clin Dermatol.

[8] Garbino JA, Naafs B, Salgado MH, Ura S, Virmond MC, Schestatsky P (2011). Association between neuropathic pain and A-waves in leprosy patients with type 1 and 2 reactions. J Clin Neurophysiol.

[9] Giesel LM, Pitta IJ, da Silveira RC, Andrade LR, Vital RT, Nery JADC, Hacker MAVB, Sarno EN, Rodrigues MMJ (2018). Clinical and neurophysiological features of leprosy patients with neuropathic pain. Am J Trop Med Hyg.

[10] Gonçalves SD, Sampaio RF, Antunes CM (2008). Occurrence of neuritis among leprosy patients: survival analysis and predictive factors. Rev Soc Bras Med Trop.

[11] Haanpää M, Attal N, Backonja M, Baron R, Bennett M, Bouhassira D, Cruccu G, Hansson P, Haythornthwaite JA, Iannetti GD, Jensen TS, Kauppila T, Nurmikko TJ, Rice AS, Rowbotham M, Serra J, Sommer C, Smith BH, Treede RD (2011). NeuPSIG guidelines on neuropathic pain assessment. Pain.

[12] Haroun OM, Hietaharju A, Bizuneh E, Tesfaye F, Brandsma JW, Haanpää M, Rice AS, Lockwood DN (2012). Investigation of neuropathic pain in treated leprosy patients in Ethiopia: a cross-sectional study. Pain.

[13] Jardim MR, Vital R, Hacker MA, Nascimento M, Balassiano SL, Sarno EN, Illarramendi X (2015). Leprosy neuropathy evaluated by NCS is independent of the patient’s infectious state. Clin Neurol Neurosurg.

[14] Mani S, Darlong J, John A, Govindharaj P (2015). Non-Adherence to steroid therapy in leprosy reaction and neuritis. Lepr Rev.

[15] Naafs B, van Hees CL (2016). Leprosy type 1 reaction (formerly reversal reaction). Clin Dermatol.

[16] Raicher I, Stump PR, Baccarelli R, Marciano LH, Ura S, Virmond MC, Teixeira MJ, de Andrade DC (2016). Neuropathic pain in leprosy. Clin Dermatol.

[17] Raicher I, Stump PRNAG, Harnik SB, de Oliveira RA, Baccarelli R, Marciano LHSC, Ura S, Virmond MCL, Teixeira MJ, de Andrade DC (2018). Neuropathic pain in leprosy: symptom profile characterization and comparison with neuropathic pain of other etiologies. Pain Rep.

[18] Rambukkana A, Yamada H, Zanazzi G, Mathus T, Salzer JL, Yurchenco PD, Campbell KP, Fischetti VA (1998). Role of alpha-dystroglycan as a Schwann cell receptor for Mycobacterium leprae. Science.

[19] Rambukkana A, Zanazzi G, Tapinos N, Salzer JL (2002). Contact-dependent demyelination by Mycobacterium leprae in the absence of immune cells. Science.

[20] Ramos JM, Alonso-Castañeda B, Eshetu D, Lemma D, Reyes F, Belinchón I, Gorgolas M (2014). Prevalence and characteristics of neuropathic pain in leprosy patients treated years ago. Pathogens Global Health.

[21] Santos VS, Santana JC, Castro FD, Oliveira LS, Santana JC, Feitosa VL, Gurgel RQ, Cuevas LE (2015). Pain and quality of life in leprosy patients in an endemic area of Northeast Brazil: a cross-sectional study. Infect Dis Poverty.

[22] Saunderson P (2000). The epidemiology of reactions and nerve damage. Lepr rev.

[23] Scollard DM, Truman RW, Ebenezer GJ (2015). Mechanisms of nerve injury in leprosy. Clin Dermatol.

[24] Silva SF, Griep RH (2007). Reação hansênica em pacientes portadores de hanseníase em centros de saúde da Área de Planejamento 3.2. do Município do Rio de Janeiro. Hansenol Int.

[25] SORRI. Monofilament aesthesiometer: Touch sensitivity testing kit. User’s manual. São Paulo: SORRI-Bauru 2008.

[26] Statistical Package for the Social Sciences SPSS Inc. Released 2007. SPSS for Windows, Version 16.0. Chicago, SPSS Inc.

[27] Toh HS, Maharjan J, Thapa R, Neupane KD, Shah M, Baral S, Hagge DA, Napit IB, Lockwood DNJ (2018). Diagnosis and impact of neuropathic pain in leprosy patients in Nepal after completion of multidrug therapy. PLoS Negl Trop Dis.

[28] Vital RT, Illarramendi X, Nascimento O, Hacker MA, Sarno EN, Jardim MR (2012). Progression of leprosy neuropathy: a case series study. Brain Behav.

